# Supplementary material for: Association between oral microbiome and seven types of cancers in East Asian population: a two-sample Mendelian randomization analysis
Source: Front Mol Biosci. 2023 Nov 21;10:1327893. doi: 10.3389/fmolb.2023.1327893 (PMC10702768; doi:10.3389/fmolb.2023.1327893)
Supplement: Supplementary file 1 [file DataSheet1.ZIP › Supplementary Materials/MR plots of saliva/saliva═╝/colorectal cancer/pheno.647_to_colorectal cancer_forest.pdf]

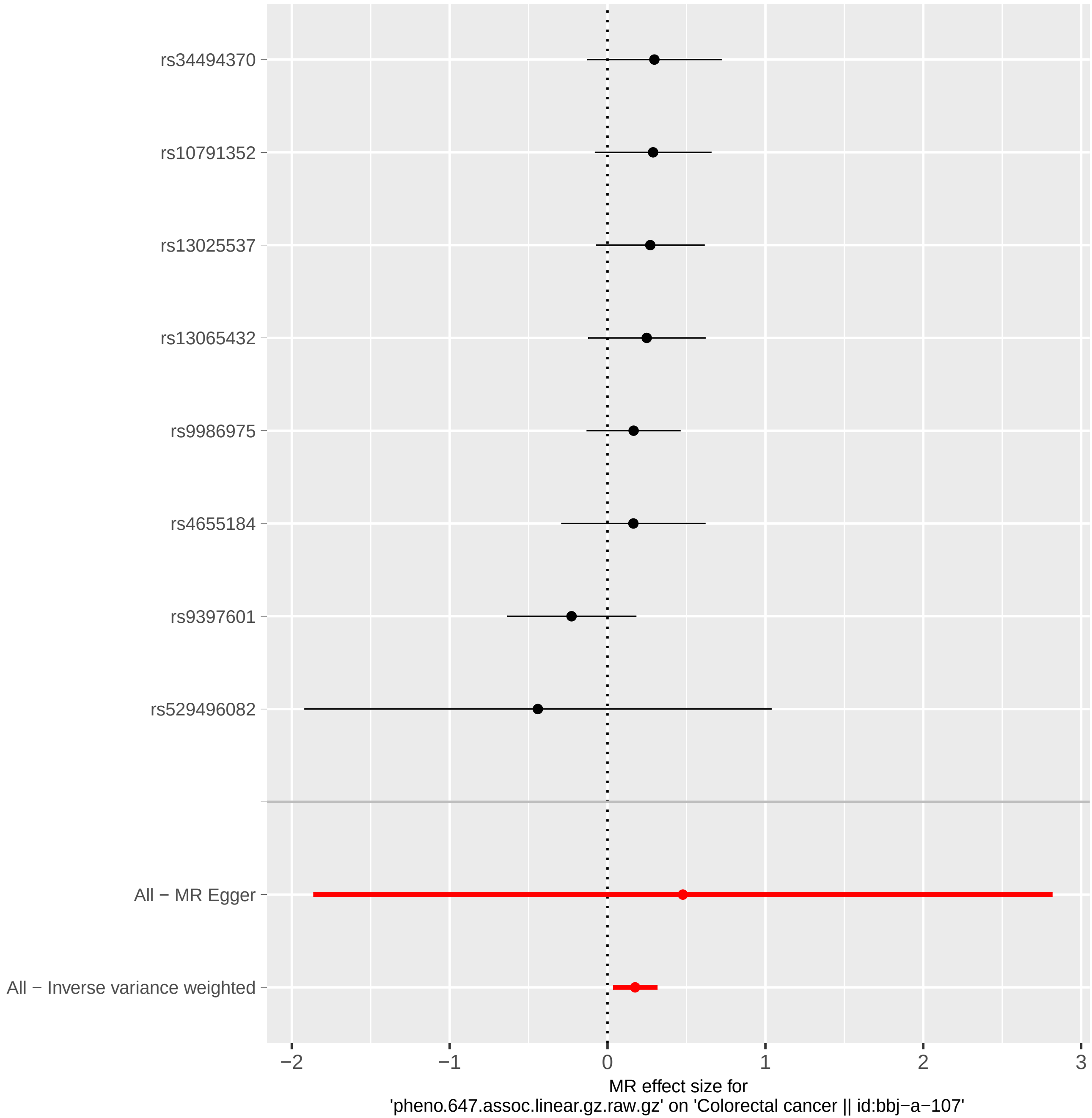

rs34494370
rs10791352
rs13025537
rs13065432
rs9986975
rs4655184
rs9397601
rs529496082
All − MR Egger
All − Inverse variance weighted
−2
−1
0
1
2
3
MR effect size for
'pheno.647.assoc.linear.gz.raw.gz' on 'Colorectal cancer || id:bbj−a−107'
